# Some Factors from Theory, Simulation, Experiment and Proteomes in the Current Biosphere Supporting Deep Oceans as the Location of the Origin of Terrestrial Life

**DOI:** 10.3390/life12091330

**Published:** 2022-08-28

**Authors:** J. W. Halley

**Affiliations:** School of Physics and Astronomy, University of Minnesota-Twin Cities, Minneapolis, MN 55455, USA; halle001@umn.edu

**Keywords:** origin of life, complex chemical systems, prebiotic chemistry, ocean trenches, prions

## Abstract

Some standard arguments are reviewed supporting deep ocean trenches as a likely location for the origin of terrestrial life. An analysis of proteomes of contemporary prokaryotes carried out by this group is cited as supporting evidence, indicating that the original proteins were formed by quenching from temperatures close to the boiling point of water. Coarse-grained simulations of the network formation process which agree quite well with experiments of such quenches both in drying and rapid fluid emission from a hot to a cold fluid are also described and cited as support for such a scenario. We suggest further experiments, observations and theoretical and simulation work to explore this hypothesis.

## 1. Introduction

It is well known that the chemistry of terrestrial life is essentially universal throughout the biosphere. That appears to imply that the successful initiation of life on earth only occurred once. That is very well known, but an implication which appears to follow quite unambiguously is less often discussed in the literature: the absence of any hint of ‘exotic’ life depending on different molecular systems appears to imply that the biochemical events leading from nonlife to life were rare events, in the sense that the probability per year of their occurence was less than something of the order of 10${}^{-9}$. That, in turn strongly suggests that a huge number of unsuccessful natural ‘experiments’ had to be performed before natural processes stumbled on a successful combination permitting Darwinian evolution to start. An ultimately successful model accounting for life’s origin will, it seems, have to take into account the rare nature of the initiating events and will therefore have to involve a process that occurred rapidly and repetitively over hundreds of millions of years.

In such a model, one will need to understand how a very large number of natural experiments could have been proceeding on early earth in order to sort through enough chemical combinations to encounter a successful one. The rare nature of several later key developments in the history of terrestrial life, such as the appearance of eukaryotes and of multicellularity, also needs to be acknowledged and taken into account to explain those events as well, but I will focus here on the earlier steps.

Taking the rare nature of the initiating event into account helps one to understand why laboratory experiments have had so little success in initiating much of any aspect of cellular life from abiogenic material despite enormous effort. It suggests that to understand the natural originating event, one needs to conceive of trial and error on a scale that may need to be hundreds of orders of magnitude larger than is feasible in any human laboratory. A very simple example of the sort of numbers involved is the well-known estimate [1,2] of the time required to form an initial genome by a random assembly of nucleotides, which results in times which are 1000 s of orders of magnitude longer than the present age of the universe. This is sometimes called ‘Eigen’s paradox’. Setting the issue of forming an initial genome aside, others suggest that the formation of a complex of interacting proteins with lifelike properties, possibly somewhat like modern prions and amyloids, is more likely. A primitive estimate illustrates that such a scenario greatly reduces the expected times to initiation of lifelike chemistry, although the times remain extremely long:

Suppose that one protein such as the one [3,4,5,6,7] in prions must be formed to start the process. I choose the prion protein (there is only one) here as an example to illustrate the orders of magnitude involved in the time required for a protein first model to generate a lifelike start. (Our models, described in Section 2, do not explicitly model that protein nor do they assume that prions initiated terrestrial life.) The PrP protein in prions is reported [4] to contain 209 amino acids. (I am not concerned here with the secondary structure, although it is central to functioning of the prion form PrP ${}^{Sc}$ of the protein.) It appears that the total number of types of amino acids chemically possible is not known. A total of at least 52 from meteorites is reported [8], and more than 1739 are listed in the norine database [9]. The task of a natural process to generate the starting protein (assuming only one will work) for initiating prion-like reproduction is then to sort through all the possible polypeptides of the needed length for the one that ‘works’ to start a Darwinian evolutionary process. The order of magnitude of the result only depends logarithmically on the number of available amino acids, and I will use the number 500 for the estimates in this paragraph. Then, there are roughly $500^{209}\approx 10^{564}$ possibilities. The corresponding numbers if the number of available amino acids is 100 or 1000 are $10^{418}$ and $10^{627}$, respectively. These numbers are smaller than the number of possible initial genomes in a genome-first model ($4^{10,000}\approx 10^{6020}$), but they are still very formidable.

Are the fluxes from ocean trenches large enough to make such a natural random search likely to be successful? The global water flux from high-temperature ‘smoker’-type emissions from trenches is roughly estimated at $10^{13}$ kg/year [10], and the concentrations of amino acids in high-temperature smokers have been estimated to be in the range $10^{-5}$ molar [11]. With a water density of 1 gm/cm ${}^{3}$, this gives about $10^{40}$ amino acids flowing through the rift system per year. If the required polymer is 200 units long, it will the take, on average in the case of 500 available amino acid types, $200\times 10^{564}/10^{40}\approx 10^{526}$ years for the needed polymer to appear assuming, very optimistically, that all the amino acids flowing through form polypeptides about 200 units long. Thus, even a rift mechanism leading to an abiogenic appearance of a protein-based initial life form seems likely to be an extremely rare event.

One way to further reduce the estimate is to suppose that there are a great many initial polymers, here assumed so far to be just one polypeptide, which will work. In the context of an amyloid model [12] for the origin of life, there might be some indication that many polypeptides would work because a substantial number (in the order of 10 to 100) of polypeptides have been found to form replicating, metastable amyloid fibrils, many of which cause various neurological diseases. However, to make the estimated average time for natural processes to form one of the needed polymers comparable to the present estimated age of the universe (or, for these purposes, the age of the earth, which is nearly exactly one-third of the age of the universe) would require that the fraction *f* of the total number of available polymers that would work be $f\approx 10^{-50}$. The fraction is small, but it means that a huge number $\approx 10^{514}$ of forms of ‘exotic’ lifelike systems would be possible. Because the fraction is so small, one may argue that if that is the resolution of Eigen’s paradox, then one would not expect to find any ‘exotic’ life on earth, and also that, in the event that lifelike systems are found on exoplanets, they will be extremely likely to be ‘exotic’, sharing very few of the specific biochemical features found on earth. That would make the task of identifying lifelike chemistry on exoplanets both qualitatively different and much more difficult than it is sometimes conceived to be.

I am referring to Eigen’s, not Fermi’s, paradox here: The latter refers to the fact that if one assumes that life initiation was NOT a rare event, then the failure, so far, to observe extraterrestrial life is paradoxical. I have argued here that the evidence suggests that the event WAS rare and therefore that there is no Fermi paradox. In the preceding paragraph, I entertained the possibility that the number of possible chemical starting points is very large. Then, one might expect some kind of lifelike chemistry on every ‘habitable’ exoplanet. However, in that case, although both Eigen’s and Fermi’s paradox would be, in a sense, resolved, the lifelike chemistry on other planets would be expected to be ‘exotic’, that is, different, and quite possibly extremely different, from that on earth. (There is a vast speculative literature on the Fermi paradox, which is not reviewed here. The present author’s perspective on these issues as of 2012 is described in [13].)

Here and elsewhere in this paper, I am avoiding any attempt at a precise general definition of ‘life’. Despite much discussion [14,15,16], there is no consensus on such a definition, and for our purposes here, we do not need one: For the terrestrial biosphere, it is clear what is meant because we have a vast trove of detailed data on the properties of ‘living’ things on earth. For extraterrestrial systems, we have extremely limited data on the chemistry happening near solid planetary and lunar surfaces, none of which has been claimed to correspond to ‘lifelike’ chemistry. For extraterrestrial systems, I will refer to our goal as the identification of ‘lifelike’ processes occurring on or near those surfaces. (I do not mean just mineral surfaces, although they may play a role in some prebiotic chemistry [17,18,19].) By ‘lifelike’, I will mean one or more generic processes or properties which have been deemed essential in one or more of the many proposed generic definitions of ‘life’. In practice, most of our work to date has focused on the property of sustained dynamic disequilibrium. The quantitative measures of the property we use are described in the next section. Disequilibrium is qualitatively similar to the property of ‘homeostasis’ as described in [16]. Sustained dynamic disequilibrium has sometimes been dismissed as ‘trivial’, but in fact, because of the second law of thermodynamics, it is far from trivial and is not easy to realize in a dense fluid. However, although that property is ‘lifelike’, I do not mean to imply that it is sufficient to establish that a system is ‘alive’. We have also studied [20] one variant of our model in which a property related to the property of ‘replication’ described in [16] is found, but that will not be discussed further here. If complex systems with the property of dynamic disquilibrium and a few other of the proposed generic features are found extraterrestrially, they are, on the basis of the preceding argument, very likely not to look very much like our terrestrial biosphere at all. If and/or when we have data on a few such systems, a more meaningful discussion concerning an appropriate general generic definition of ‘life’ will be possible.

I infer from these considerations that the determination of the initial chemical steps in the origin of life is a less critical question than the question of how those steps could have taken place, given that random processes which failed to produce lifelike chemistry would have been overwhelmingly more likely. I therefore suggest that a high priority is to find mechanisms for extremely rapid natural experimentation taking place in parallel over hundreds of millions of years. The general idea that many natural experiments are required is not new, but much work in this field emphasizes how slow the process must have been, whereas here, I emphasize that given the enormous natural search task, the processes had to be extremely fast at the molecular level. In the next sections, I will review some recent simulation and experimental work which suggest that the rapid quenching of hot fluids containing large amounts of biomonomers after a suitable incubation time may be the most likely process for achieving the needed massive parallel processing, such as in well known models of abiogenic evolution in the fluids emitted from ocean rifts [21]. Several ways that such quenching could have happened on early earth have been proposed, for example in hot springs or impact craters [22] as well as in ocean rifts. In view of the centrality of the need for massive processing, however, ocean rifts, which have continuously quenched very large volumes of hot fluid over hundreds of millions of year, seem at least in that respect the most favorable. Ocean rifts have other favorable features, some of which are discussed in [21]. For example, the reactions near the ocean floors would be relatively protected from ultraviolet light and high-energy cosmic rays that could interrupt the low energy chemical development required, whereas it seems less obvious that such protection would be likely to be present to the same degree in hot springs.

However, here, we emphasize another advantage of the quenches of hot fluids emerging from within the earth into cold ocean water: namely, the large volumetric flow of fluid which increases the chance of a successful initiation of lifelike chemistry. We envision the importance of a quench (which also occurs in hot springs) as follows: Proceeding at a temperature near ambient, covalent bonds such as peptide bonds have very long lifetimes as discussed further below, and the chemical sorting that would produce many combinations of monomers (amino acids in the protein case) would be extremely slow. On the other hand, at high enough temperatures, the covalent bonds will break and form, on average, much more quickly, and more polypeptides will be sampled in a ‘dynamical chemical network’ [23]. However, the instability produced by the frequent thermal breaking of covalent bonds would not be favorable to the development of lower energy processes of prebiotic evolution that use those polymers as building blocks. Thus, one needs a ‘sampling step’ in which each combination of prospective biopolymers produced at high temperatures is stabilized and allowed to evolve at low temperatures. That is quite precisely what happens in a quench when the thermal bond breaking is frozen out as the system goes quite suddenly from a hot stage to a cold one, as it emerges from the ocean rift into the cold ocean water. To freeze the high-temperature distribution of polymer lengths so that it is retained in the low-temperature, quenched, phase, the quench must occur faster than the (adiabatic) rate, which would allow the covalent bonding to completely equilibrate to the low-temperature equilibrium distribution during cooling.

In this paper, I review the analytical and computational efforts of my group over the last ten years to obtain insight concerning how nature might have produced the rare event leading to life on earth and possibly other rare events leading to lifelike systems extraterrestrially. It is not a review of the entire literature on the ocean trench model for the origin of life or of other computational models intending to provide insight into the origin of terrestrial life.

## 2. Results

I hope I have convincingly reiterated the argument that the process leading to life’s origin was extremely unlikely over the timescale set by the age of the earth, but we would like to know more quantitatively how (un)likely it was. For that, one needs some more specific ideas concerning where and by what detailed chemical processes the initiation took place. If a model for those processes is too chemically specific, it may miss some essential generic features which are promising because the chemical details embedded in the model obscure the promise. On the other hand, if the model is too coarse grained, it is likely to be uninformative. In polymer science, scientists navigate between those two dangers by coarse graining of the atomic-scale description [24]. Formally, the coarse graining can be mathematically carried out by convoluting the variables of the original atomic description with a smoothing function, such as a Gaussian. However, more commonly, some physically plausible assumptions about the essential features are made in order to specify a coarse-grained model. For example, early on, polymer chemists used a ‘united atom’ model of hydrocarbon polymers by treating the carbon atoms and the hydrogen entities bonded to them as an entity located at one point in three-dimensional space. Similarly, biochemists use models of biopolymers in aqueous solution which treat the water as a continuum (‘implicit water’) and the motions and positions of the monomers in the biopolymers with atomic specificity.

In addition to the practical computational advantages, coarse graining can reveal features which may be obscured by the mass of noisy detail in a more fine-grained description. If features appear in the coarse-grained description of a system which were obscured in a more detailed description, statistical physicists speak of ‘emergence’ [25,26]. One may regard terrestrial life itself as an emergent phenomenon in that sense, since it is difficult to discern the essential features of life by regarding, for example, a molecular dynamics simulation of protein motions on angstrom and picosecond scales. (However, the problems in attempting to make such a statement quantitative are outlined in [26].) The distinction between fine-grained and coarse-grained descriptions is qualitatively similar (but not isomorphic) to that between biological descriptions in terms of genotype (an atomic-scale description) and phenotype(usually a more coarse-grained description of structure or behavior), respectively. There is another advantage to coarse graining if one is interested in the likelihood of extraterrestrial lifelike systems, possibly on exoplanets or moons: Given the enormous combinatorial space available, it is much more likely than not that such lifelike exobiological systems, if ever found, will be exotic in the sense that the basic biochemistry will be very different from the terrestrial one. Such possiblities will be missed in models which are too chemically specific. Thus, coarse graining avoids some aspects of terracentricity.

The coarse-grained descriptions which we have been using in my group in efforts to gain insight into the origin of life were initially inspired by the work of Stuart Kauffman and coworkers [27,28,29], who introduced a coarse-grained model for the origin of life in the 1980s. In it, there were ‘monomers’ of an integer number *b* of different types which were allowed to link together to form ‘polymers’ through a process termed ‘ligation’ and to become unlinked through the reverse process of ‘scission’. A parameter *p* in the range [0,1] was introduced which specified the likelihood that any given reaction among all the possible ones would actually occur. There was no spatial component: one could say that the model described a ‘well-mixed’ reactor. There was also no temperature and no energy. By starting with a finite set of ‘monomers’ and ‘dimers’, Kauffman et al. built a set of reaction networks and then, by randomly assigning rates to the reactions included, followed the dynamics stochastically by numerical simulation for each one. They found that for essentially all values of *p*, the populations of polymers grew without limit as long as the population of monomers and dimers was maintained (one could say by ‘feeding’). The results were regarded as suggesting that lifelike properties could be expected to appear quite readily on earth and elsewhere, which is in contrast to the discussion in the introduction to this paper.

In our first extension of the Kauffman work [30], we noted that, without extending the model, one could compute the combinatorial entropy of the states resulting from the dynamic simulations and compare it to its maximum possible value which, since the model was essentially working at infinite temperature, would be the value expected in thermodynamic equilibrium at that temperature. When we counted the fraction of times that the dynamics led to an equilibrium state by that criterion, we found that the dynamics led to equilibrium most of the time. Only a small fraction, which we interpreted as having the lifelike property of disequilibrium, was produced by the dynamics. The others were growing in population, but they were just adding molecules to a system in equilibrium, and we interpreted them as not lifelike. One could ask, in that context, why all the final states were not in equilibrium since it is predicted by the second law of thermodynamics that dynamic processes always increase the entropy. However, the states we found that were not in equilibrium were kinetically trapped by the sparsity of the network in a kind of cul de sac of the chemical space. They were taking a longer time to escape such ‘kinetic traps’ than the length of the computer run and were thus in a metastable disequilibrium state, as living things are.

Although we found that interesting, the model left out a great deal. In particular, there was no energy, no temperature and no spatial dependence (as well as no detailed chemical description of the monomers, but we regarded the latter as an advantage for the reasons cited above). There were several stages [20,31,32,33,34,35,36] in the effort to take account of these features, but I will skip the intervening steps and describe our most recent efforts in which, as a kind of bonus, we found a way to interpret the somewhat mysterious Kauffman parameter *p* in terms of more directly accessible experimental quantities. We retain a description of the available reactions as the ligation and scission of polymers of monomers of *b* species. It should be noted that this assumption is open to serious question if one wants, as we do, to use our results to model what might happen during the quenches taking place as fluids are emitted from ocean rifts. That is because although the ligation reactions leading to polypeptides and nucleic acids are endergonic and high temperatures can therefore enhance peptide bond formation for example, the formation of the monomers themselves, amino acids in the case of peptides, is believed to be exergonic so that the monomers themselves would not survive [21]. On the other hand, we noted above [11] that amino acids are detected observatonally in the fluids emitted, and studies of the effects of hydrogen in stabilizing amino acids in hydrothermal environments [37] suggest that amino acids survive in black smokers.

To take account of energetics, we introduced a binding energy Δ which is the energy difference between two unbonded monomers and the same two monomers when bonded. If Δ<0, it means that the bonded system is at higher energy than the unbonded one, as is the case for both peptide bonds in proteins and for nucleic acids in RNA and DNA in the absence of enzymes. In our most recent work, we therefore consider only that Δ<0 case and also introduce an activation energy $\Delta_{a}$ which controls the rate of scission. Unlike Δ, $\Delta_{a}$ takes different values for each reaction, as selected from a Gaussian distribution with a mean $\bar{\Delta_{a}}$ fixed by experiment. In particular, in our application to polypeptides, we fix $\bar{\Delta_{a}}$ to be the measured average activation energy for the hydrolysis of the glycine–glycine peptide bond in water [38]. The two energy parameters are used to fix the rates in the dynamics simulation. The ‘forward’ (scission) rate for a reaction 1→2+3 is given $vk_{d}n_{1}$ and the reverse rate is $vn_{2}n_{3}/k_{d}$, where $k_{d}^{2}=\bar{n_{2}}\bar{n_{3}}/\bar{n_{1}}$ the line over the *n* ’ *s* in the definition of $k_{d}^{2}$ indicates the equilibrium distribution of the species. The factors *v* take the form $v=e^{-\Delta_{a}/k_{B}T}$ and the $\Delta_{a}$ values are drawn during network formation from a Gaussian distribution centered on the average activation energy as experimentally determined. The physical rates are interpreted to be $f_{a}v$, where $f_{a}$ is the measurable prefactor, sometimes termed the ‘attempt rate’, in the rate constant for scission, or in the case of the polypeptides, the hydrolysis of the peptide bond. With that parameterization, the model as written mathematically and coded is expressing time in units of $1/f_{a}$, and we can convert that to physically measured time in the laboratory or observation by multiplying computed rates by $f_{a}$ and times by $1/f_{a}$. The factors $k_{d}$ guarantee that the system is being driven toward equilibrium in accordance with the second law, although we are interested in cases in which it stalls in a metastable lifelike state before it gets there. The width of the Gaussian is another parameter. We think of the Gaussian distribution as arising from fluctuations in the activation energy arising from a heterogeneous environment, and in the case of peptides, we found very little indication of an appropriate value for its width σ in the literature. We obtain an order of magnitude estimate from the data in [38] on glycine–glycine hydrolysis where the activation energy is reported to vary substantially for glycine–glycine bonds in a few different polypetides.

In this model, we have taken account of some missing features, but now every possible reaction is allowed, so we need to understand how kinetic trapping can occur. That is because the experiments, natural or made by humans, take a finite time say of order $t_{expt}$. Even at the average activation energy, the mean time for hydrolysis to occur in peptides can be as much as a century, and the Gaussian distribution allows the possibility of much longer times. Thus, those reactions for which the rate $f_{a}v<1/t_{expt}$ will not occur during the experiment and can be left out of the networks. Therefore, we can predict, on the basis of parameters which are experimentally known (within some estimatable uncertainties), the value of the fraction $p_{eff}$ of the reactions which should be included in the network, just as they were in the original Kauffman model. Working out the details [36], the parameter $p_{eff}$ can be expressed in terms of combinations of the experimental quantities $\xi =\bar{\Delta_{a}}/\sigma$ and $T_{c}=\bar{\Delta_{a}}/\ln(t_{expt}f_{a})$ as described in detail in Section 2.2 below.

It turns out that the model predicts that a swift quench from above the temperature $T_{c}$ to a temperaturre below $T_{c}$ will take the system from a state in which nearly all possible reactions involving the breaking and forming of covalent bonds are occuring to one in which they almost all stop abruptly. (‘Swift’ means fast compared to the rate at which the system equilbrates to the temperature of its external bath.) This is one of several indications that suggest that the original peptides were formed in a high-temperature environment and then quenched. Before describing that model result in more detail, in the next section, I review more model-independent direct experimental evidence, which we found [32] in data on the proteomes of prokaryotes indicating that quenches may have led to the formation of proteins.

### 2.1. Evidence from Biodata for an Origin in a Quench

The first indication of a quench origin for terrestrial life which we noted came from a study which we carried out [32] of length distributions in proteomes of prokaryotes, where the length of each protein was the number of amino acids in the chains of proteins. We analyzed data on the proteomes of all the prokaryotes listed in the KEGG database [39] in 2018. The motivation for the study was not initially to establish quenching as a mechanism for the origin of life but to test our hypothesis that a measure that we had been using to detect lifelike systems in our simulations did indeed distinguish real living systems from nonliving ones. To clarify the meaning which we attribute to one of the results of [32] in the context of the present paper, the definition of the measures used there is reviewed here. It should be emphasized that the results of reference [32] were obtained without use of any of the details of the network and dynamics models which we used for simulations in references [20,30,31,35] as described in the next section, although we used the same measure to analyze the simulation results in some of those papers that we used in [32] to analyze experimental results from the proteome study. We assumed in [32] that the bond energies (parameterized by a parameter Δ<0 as discussed in the previous section) of all the possible amino acid pairings in the proteomes were the same and that no other energies were relevant in establishing the equilibrium state of the proteome system with regard to its polymer length distribution. That is a kind of coarse graining in the energy description as further discussed in [35]. Using that parameterization, the data provide the relevant numbers $\{N_{L}\}/V$ and e=E/V where *V* is the volume, $N_{L}/V$ is the number of proteins per unit volume having (amino acid) length *L* and *E* is the total bonding energy. The energy of a protein of length *L* is −(L−1)Δ in such a description, and the total energy *E* per amino acid of a state of the proteome characterized by the distribution $\left\{N_{L}\right\}$ is $-\sum_{L}\left(L-1\right)\Delta /N$ where $N=\sum_{L}N_{L}$.

Now, we suppose that the system is in contact with a thermal bath at temperature *T*. We can define two different equilibrium distributions for the system, one in which the system is in thermal equilibrium with the bath and a second in which the system is self-equilibrated but has not equilibrated to the bath. This is a textbook distinction in physics [40], but it is less familiar in the context of origin of life studies, so I will briefly dwell somewhat further on it. The first form of equilibrium will be attained in a nonliving system (and even in a living system that has died) after enough time has passed for the system to equilibrate to an external bath. Because the bath might be outside the system and only accessing it through a two-dimensional surface in space, that equilibration can take a long time. When it does, one has the familiar canonical distribution reviewed below. The second, self-equilibration can occur faster, although it only does so in so-called ergodic systems. The length distributions in that second equilibrium state have the same form as they do in the first kind of equilibrium, but the effective temperature is determined differently and depends on *E* but not on the external *T*, as seen in the equations reviewed below. One can say in textbook language that the second distribution is microcanonical because the energy and not the outside temperature is fixed, but it is not described by a δ function describing the energy condition. INstead, it is described by a distribution of the canonical form but with an effective temperature determined from the total energy. The description of this isolated second type of equilibrium state in terms of a delta function and the description in terms of a canonical distribution with a temperature determined from the total energy *E* turn out to be identical in the thermodynamic limit of large volumes and large particle numbers [40]. That thermodynamic limit will be very nearly reached in most macroscopically observed systems of interest here. The effective temperature of the isolated state can be different from the bath temperature if the system has not been in contact with the bath long enough. If it has been in contact with the bath for a very long time, then the two forms of equilibrium are expected to be the same. The relevance to a quench is that a system of amino acids in contact with a high-temperature bath may attain equilibration of the first type because the processes are fast at high temperature but then take a very long time to equilibrate to the cold bath into which it is plunged by the quench. Then, the distribution of polymer lengths in the system will reflect the temperature of the hot bath and not of the bath with which it is currently in contact. In that way, we may be able to read something about the thermal history of the system from its length distribution. That is how we proposed to interpret the results of reference [32].

I provide a few details of the determination of an equilbrium length distribution from [32] to indicate the meaning of the measures of disequilibrium which we used: We denote the total number of polymers *N* in a sample by $N=\sum_{L=1}^{l_{max}}N_{L}$. However, in contrast to the situation in the dynamic simulations described in [31], the input data for the calculation of equilibrium distributions are not *N* and *E* but the volumetric polymer concentration ρ=N/V where *V* is the solution volume and the volumetric energy density e=E/V. To take entropic account of the dilution of the experimental sample, we introduced a microscopic length $l_{p}L^{\nu }$ where $l_{p}$ is the (microscopic) polymer persistence length and ν is a dimensionless index, which would be 1/2 for a random walk. For denatured proteins, ν has been determined experimentally [41] and is close to the value expected for a self-avoiding walk. We then modified the expression for the entropy used in [31] to take account of the number of ways to distribute *N* polymers in a volume *V* giving entropy $S/k_{B}=\ln W$ with

$$W=\sum_{L}\frac{(N_{L}+G_{L}-1)!}{N_{L}!\left(G_{L}-1\right)!}$$

and $G_{L}=b^{L}V/v_{L}$ and $v_{L}=l_{p}^{3}L^{3\nu }$. *b* is the number of monomers available for inclusion in the polymers in the system. The expression is identical to the one used in [31] except for the factor $V/v_{L}$ in the degeneracy $G_{L}$.

Proceeding in the standard way to maximize the entropy under these constraints, we have when both energy density e=E/V and polymer number density ρ=N/V are fixed that the values $\bar{N_{L}}$ of the populations that maximize this entropy are

(1) $$\bar{N_{L}}=\frac{G_{L}-1}{\exp(-\beta (e,\rho )\mu (e,\rho )-\beta (e,\rho )\Delta (L-1))-1}$$

Here, the parameters β(e,ρ) and μ(e,ρ) are determined from the total energy density *e* and polymer number density ρ=N/V by the implicit equations (with (4))

(2) $$e=-\left(1/V\right)\sum_{L=1}^{l_{max}}\left(L-1\right)\bar{N_{L}}\Delta$$

and

(3) $$\rho =\left(1/V\right)\sum_{L=1}^{l_{max}}\bar{N_{L}}$$

We use the definition of $G_{L}$ and define $v_{p}=l_{p}^{3}$ to write these relations as

(4) $$\bar{N_{L}\left(v_{p}/V\right)}=\frac{b^{L}/L^{3\nu }-\left(v_{p}/V\right)}{\exp(-\beta (e,\rho )\mu (e,\rho )-\beta (e,\rho )\Delta (L-1))-1}$$

and

(5) $$ev_{p}=-\sum_{L=1}^{l_{max}}\left(L-1\right)\bar{N_{L}\left(v_{p}/V\right)}\Delta$$

and

(6) $$\rho v_{p}=\sum_{L=1}^{l_{max}}\bar{N_{L}\left(v_{p}/V\right)}$$

These are in dimensionless form, which is convenient for solving for β(e,ρ)μ(e,ρ) and β(e,ρ)Δ numerically because they do not involve macroscopically large numbers. 1/β((e,ρ) is Boltzmann’s constant times the temperature fixed by self-equilibration and the given energy and particle density. μ(e,ρ) is the corresponding self-equilibrated chemical potential. $\rho v_{p}$ is the estimated (fractional) number of polymers in a cube of volume $v_{p}$ and is a small number. The term $v_{p}/V$ on the right-hand side of (1) is in all cases much less than $b^{L}/L^{3\nu }$ and is dropped in the numerical analysis. This is the distribution arising from the second kind of equilibrium discussed above in which the system is either isolated from any bath or, more often, has not had time to equilbrate to an external bath. We have sometimes [31] referred to this equilbrium as ‘isolated’. There is no reference to the temperature of an external bath.

To obtain the first kind of equilibrium distribution, we solved (3) for μ where we replaced β(e,ρ) and μ(e,ρ) by $1/k_{B}T$ and μ(T,ρ), respectively, obtaining μ(T,ρ) with a fixed value of *T*. We used values of the ambient temperature in determining that first kind of equilibrium distribution and made no use of (2). In a quench, the ambient temperature is the high temperature of the fluid before the quench and the low temperature of the fluid after the quench. That first kind of equilibrium distribution then describes the expected distribution when the system has had time to equilibrate to an external bath. In each case, we use (1) to evaluate the polymer length density distributions expected in those two equilibrium states. After a quench, if it is fast, the first kind of equilibrium is often not achieved by the system for a long time. Note that operationally ‘fast’ here means fast compared to the rate at which the system equilibrates to the lower external temperature in the quenched state.

Because the systems of interest are not necessarily (and in fact are often found not to be) in either kind of chemical equilibrium, we used the experimentally observed values of the polymer length densities $N_{L}\left(v_{p}/V\right)$ to evaluate Euclidean distances in the space of values of sets $\left\{N_{L}v_{p}/V\right\}$ between the actual population set $\left\{N_{L}v_{p}/V\right\}$ and the ones corresponding to the two kinds of equilibria given by (1) with β(e,ρ), μ(e,ρ) in the isolated case and with a fixed β and μ(T,ρ) in the case in which the system is equilibrated to an external bath. Thus, we define two Euclidean distances $R_{L}$ and $R_{T}$ in the $l_{max}$-dimensional space of sets $\left\{N_{L}\right\}$ which characterize how far the system of interest is from the two kinds of equilbrium:

(7) $$R_{L}=\sqrt{\sum_{L}{\left(v_{p}/V\right)}^{2}{\left(N_{L}-\bar{N_{L}\left(\beta \left(e,\rho \right),\mu \left(e,\rho \right)\right)}\right)}^{2}}/\left(\sqrt{2}v_{p}\rho \right)$$

for distance from the locally equilibrated state and

(8) $$R_{T}=\sqrt{\sum_{L}{\left(v_{p}/V\right)}^{2}\left(N_{L}-\bar{N_{L}(\beta ,\mu \left(T,\rho \right)}\right){)}^{2}}/\left(\sqrt{2}v_{p}\rho \right)$$

for distance from the equilibrium state with the external bath. $R_{L}$ and $R_{T}$ are, respectively, measures of the distances from the two kinds of equilibrium discussed above. A similar measure in the context of origin of life studies was suggested in [33].

**Figure 1:**
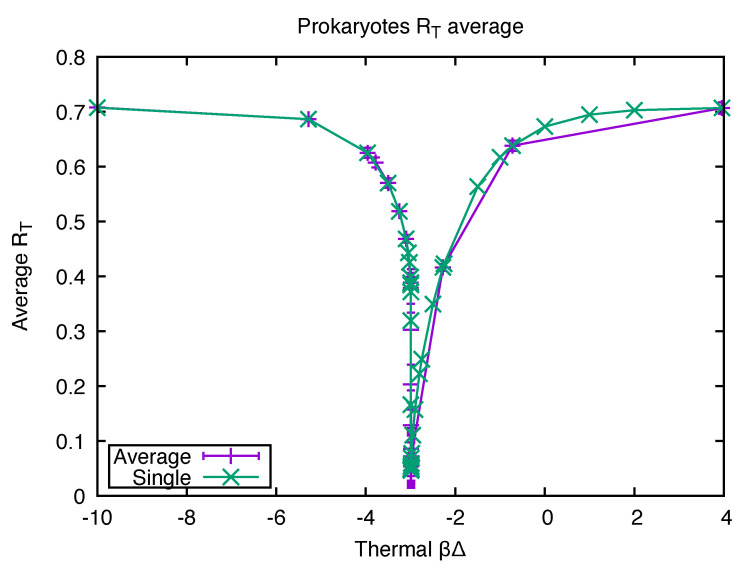
$R_{T}$ as a function of βΔ for one prokaryote (KEGG code ebw, green crosses) and the average values over the entire KEGG database of 4555 prokaryotes (purple plusses). The error bars (purple) indicate the standard deviation over the full data set. The variable βΔ is the bond energy Δ divided by $k_{B}T$ where *T* is the assumed ambient temperature. The graph shows that the proteome has a length distribution at a temperature close to equilibrium at about 400,000, although most of the prokaryotes commonly live at much lower temperatures.

In [32], we reported on the use of those measures in several ways, but here, I focus on some of the results for the proteomes. With a given polymer length distribution, we could plot $R_{T}$ as a function of the temperature assumed in the equation for $R_{T}$. ($R_{L}$ does not depend on the external temperature.) Most of the prokaryotes considered currently live in an environment with a temperature close to ambient, but we explored how the $R_{T}$ value varied with different assumed external bath temperatures. We show a typical result for one of the proteomes and also the average $R_{T}$ for all 4555 of them in Figure 1. To our surprise, there was a very sharp drop in the calculated value of $R_{T}$ as a function of *T* at a temperature quite close to the boiling point of water. At the minimum, the $R_{T}$ value is essentially identical to the $R_{L}$ value, and the effective temperature of the isolated equilibrium was also found to be near the same temperature at which the dip in $R_{T}$ was observed.

We interpreted this to suggest that the original proteins in the prokaryotes were formed at the high temperature around the boiling point of water and were then quenched to a cooler temperature which froze out most of the reactions which broke or formed peptide bonds so that the higher temperature length distribution was frozen into the subsequent evolution of the system. The data leading to Figure 1 thus suggest that all 4555 proteomes arose from a collection of proteins which formed at the same temperature. Many events which we are not attempting to model here occurred after that initial collection of proteins was formed (including, for example, the introduction of a genetic code, the appearance of the ribosome, and the adaptation of various prokaryotes to different environments including high-temperature ones).

There are issues associated with time scales in this interpretation. Peptide bonds in water can have reaction constants for hydrolysis, leading to average times for bond breaking at ambient temperatures of up to centuries. However, the suggestion requires that the length distributions in the prokaryotes survive not centuries but rather more than 3 billion years. To avoid a contradiction, we suggest that once a sufficiently promising system was quenched by chance, the transition to a lifelike system evolving and growing by natural selection might occur much more rapidly than equiibration to the external bath. For example, in the case of polypetides, that would require that the initiation through Darwinian evolution of processes such as autocatalysis to replace damaged polymers and protect the nonequilibrium state would need to occur in a time less than the order of a century. The resulting nonequilibrium steady state could then be self-sustained by those evolved processes, and the associated polymer length distributions also could be preserved for much longer times, possibly up to billions of years. If the starter protein was like PrP ${}^{Sc}$, for example, that might be conceivable, since prion reproduction [34] does not appear to make much use of the elaborate reproductive machinery of modern cells, and only a few steps might be required.

### 2.2. Evidence from Theory and Simulation for an Origin in a Quench

**Figure 2:**
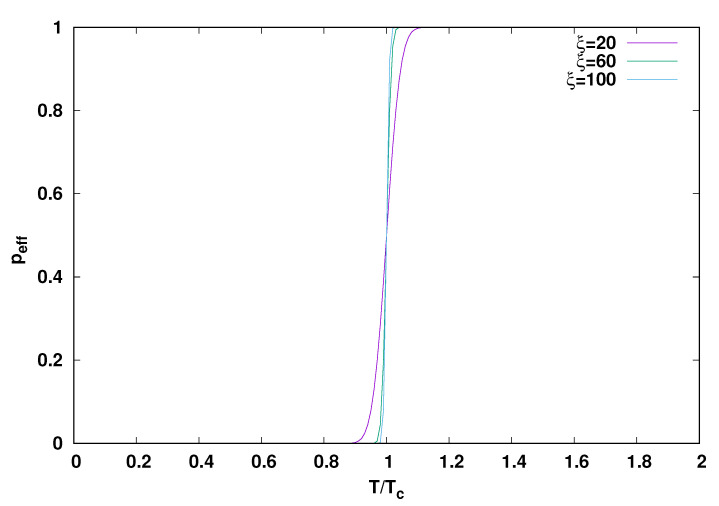
$p_{eff}$ as a function of temperature for various values of ξ using Equation (10).

Turning to theory and simulation, we show the form of the effective fraction $p_{eff}$ of the possible scission and ligation reactions briefly described at the end of the Section 1 as predicted by the model of reference [35] in Figure 2. The model provides an analytical expression for $p_{eff}$ which depends only on the combinations of experimentally determinable quantites $\xi =\bar{\Delta_{a}}/\sigma$ and $T_{c}=\bar{\Delta_{a}}/\ln(t_{expt}f_{a})$. $T_{c}$ is a temperature, and the figure shows that for parameters suitable for the description of peptides, $p_{eff}$ undergoes a very sharp transition from nearly one to nearly zero when *T* passes from above $T_{c}$ to below it. (The analytical form or $p_{eff}$ in terms of ξ and $T_{c}$ is determind by evaluation of the integral

(9) $$p_{eff}=\int_{f_{a}t_{expt}}^{\infty }\left(dP/dv\right)dv$$

where dP/dv is the probability distribution of the values of *v* determined from the assumptions cited above. The result is

(10) $$p_{eff}=\frac{erf\left(\xi \right)-erf(\left(1-T/T_{c}\right)\xi )}{erf(\xi )+1}$$

where erf is the error function [42]. (Please see [36] for details. ) $T_{c}$ is about 370,000 in the peptides and is independent of the poorly known parameter σ.

The sharp change from 1 to 0 in $p_{eff}$ illustrated in Figure 2 and predicted by the detailed model of reference [36] thus occurs at a temperature quite similar to the one at which there is a relatively model independent dip in $R_{T}$, which we found in [32] to occur in the proteomes. We suggest that the model thus provides additional support for an origin of life scenario such as the one described in Section 1 in which a quench took the system from a $p_{eff}$ of nearly 1 at a temperature above $T_{c}$ to a value of *T* below $T_{c}$ with a $p_{eff}$ of nearly zero, at which point spontaneous hydrolysis and peptide bond formation almost stopped. The temperature of the dip in $R_{T}$ arose from the proteome data, but the sharp shift in $p_{eff}$ depends on just a few parameters: The average value $\bar{\Delta_{a}}$ of the activation energy for peptide bond hydrolysis, the ‘experimental’ time $t_{expt}$ which the system spends at high temperatures before quench and the prefactor $f_{a}$, interpreted as a frequency, in the Arrenhius relation for the hydrolysis rate. The detailed relation is

(11) $$T_{c}=\frac{\bar{\Delta_{a}}/k_{B}}{\ln(t_{expt}f_{a})}$$

The most poorly known quantity here is $t_{expt}$. ($T_{c}$ is independent of the parameter σ.) We have some knowledge of $t_{expt}$ in laboratory experiments briefly described below. In the case of smokers from ocean trenches, the relevant time is presumably the average time which the fluids spend in the high-temperature part of the circulation pattern, which takes low-temperature water through the porous crust of the ocean floor near a ridge to a horizontal path near the hot magma of the interior, after which it rises through the rift to emerge in the smokers [10,43]. These events, while believed to occur quite generally, are reported to be extremely heterogeneous in their time scales. However, time scales of the order of 1 to 10 years are typically suggested, even though some processes are much faster. Solving Equation (11) gives $t_{expt}=f_{a}^{-1}exp(\bar{\Delta_{a}}/k_{B}T_{c})$, and using $\bar{\Delta_{a}}/k_{B}\approx 1.2\times 10^{4}$ K and $f_{a}\approx 1.3\times 10^{7}$ s ${}^{-1}$ as extracted from the data in [38] for peptide hydrolysis rates, we find $t_{expt}\approx 0.3$ years using $T_{c}=370$ K. Thus, this scenario appears to be roughly consistent with what is currently known. However, the values of the activation energies reported in [38] vary by as much as a factor of 2 depending on what pair of bonded amino acids one is hydrolyzing, so this conclusion is tentative.

The measurements of amino acid concentrations in the emissions from smokers in ocean rifts reported in [11] and cited earlier provide another piece of possibly relevant information: The concentrations of peptides were measured in a way that permitted the concentration of all polypeptide molecules including monomers to be measured as well as the concentration of amino acid monomers alone. The former was much larger than the latter, suggesting that the amino acids were mainly in bonded polypeptides, although a detailed length distribution was not found. That might suggest support for an origin of life scenario, since long polypeptides would be needed, but it might be in contradiction with laboratory experiments discussed below where many more monomers than polypeptides were found. One possible explanation is that the high-temperature phase of the quench in the laboratory experiments was much lower than the average temperature of the fluids in the relevant natural ocean rift experiments, and the higher temperature would lead to the formation of more long polymers, which would be retained upon quenching. Some of the fluid temperatures in the high-temperature phase of the quenches occurring in ocean rifts are reported to be much higher (up to nearly 600,000) than those in the reported laboratory experiments (usually around 373,000).

### 2.3. Comparison of Model Predictions with Laboratory Results of Quenches

Laboratory experiments to determine whether quench-like processes could result in enhanced polypeptide synthesis from monomeric solutions of amino acids have been reported by groups led by Matsuno [44,45,46] and somewhat less directly by John Yin [47,48,49]. Both groups observed polypeptide formation, although the numbers of polypeptides were small. The Matsuno et al. experiments directly modelled aspects of the proposed scenario in ocean ridges: Hot solution was forced through an orifice into a cold bath and then recirculated to be heated again. The experiment differed from the envisioned oceanic one because the dwell times were much shorter—of the order of a minute compared to dwell times in the hot part of the cycle in an ocean trench estimated, as reviewed above to be of the order of a year or more. As seen in Equation (11), the critical temperature above which we expect rapid polymer formation depends inversely on the logarithm of the dwell time $t_{expt}$, suggesting a significantly higher value of $T_{c}$ in the Matsuno experiments than in the ocean trenches. Thus, it is possible that the hot phase in the Matsuno cycle was at or below $T_{c}$ where polypetide formation would not be significantly enhanced. Furthermore, because the ocean ridge dwell times are believed to be longer, the predicted $T_{c}$ from Equation (11) is lower, increasing the likelihood that ocean ridge quenches would have hot phase temperatures above $T_{c}$.

**Figure 3:**
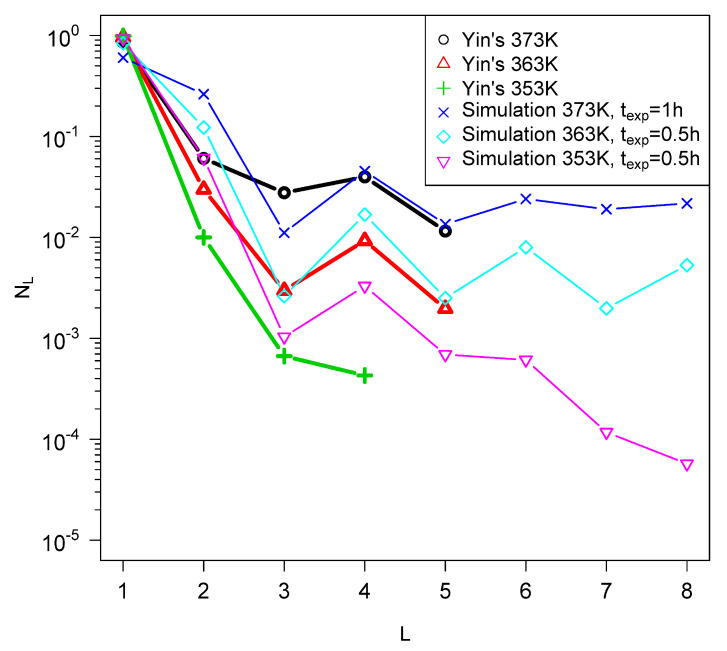
Comparison of the length distributions reported from reference [48] on drying experiments with analine–glycine mixtures with results from simulated quenches. The value of σ was assumed to be the same for all the experiments but was fitted. Parameters were $\Delta_{a}=99.7$ kcal/mol, $f_{a}=5.96\times 10^{-}7$ s ${}^{-1},\sigma =0.13\Delta_{a})$.

**Figure 4:**
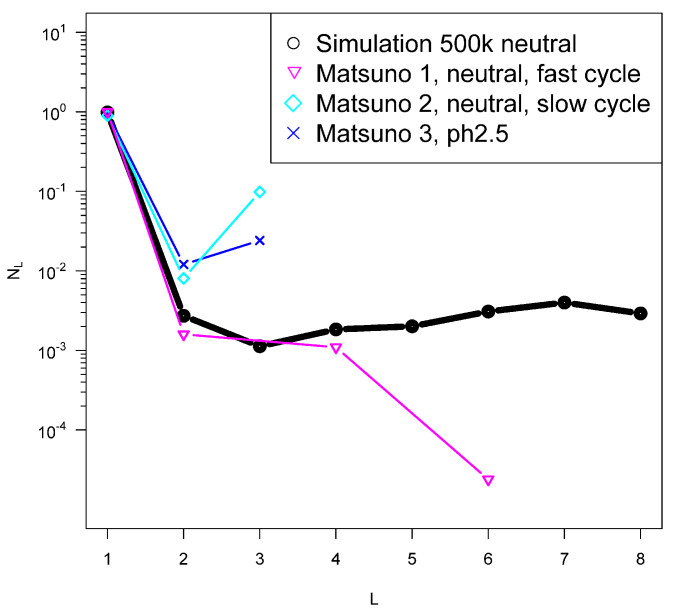
Similar to Figure 3 for data reported from quenches from hot to cold water in [44]. The black simulation was obtained with $\Delta_{a}=99.7$ kcal/mol, $\sigma =0.13\Delta_{a}$ and $p_{eff}=0.5$. We can infer from (10) that the high-temperature phase in these experiments was at temperature close to 373 K.

I show some preliminary attempts to fit our model to the data of the Yin and Matsuno groups in Figure 3 and Figure 4. Qualitative features are reproduced with reasonable parameters, but this is hardly a proof that the model exactly describes the processes taking place in these experiments. However, it is clear that the numbers of polypeptides produced is small compared to the monomers, which is consistent with the predictions of the model and in contrast to the limited information from ocean trenches as discussed above. It is of some interest that the model reproduces the even–odd oscillations with respect to *L*, which are observed in both sets of experiments, although more markedly in the Yin et al. results. We have also formulated a simplified version of the model for the case in which there are a lot more monomers than polymers, which reproduces very similar even–odd oscillations.

## 3. Discussion and Conclusions

As reviewed in [21], the idea that life may have originated on earth in the emission of hot fluids from ocean ridges is not at all new. Here, I have reviewed aspects of our work that explore the issues associated with that hypothesis from a somewhat novel perspective, which we believe contributes some additional support to the idea. In particular, we are not aware of other work that has used the polymer length distributions in existing organisms to infer the temperature during the time when the initial synthesis of biopolymers took place, as reviewed briefly here and in detail in [32]. Our models have extended the work of Kauffman to permit an interpretation of the network parameter *p* in terms of measurable quantities including the time the fluid spends at high temperature and the distribution of activation energies for bond formation. We find evidence in the models of a sharp nonequilibrium transition from long polymers and a dense reaction network to a sparse reaction network populated by a nonequilibrium population of long polymers in which the long polymers retain a kind of memory of their thermal history at low temperatures after a quench.

Some of these results might be further tested experimentally without extreme difficulty. For example, our analysis of the experiments of reference and [44,45,46,48] suggests that the high temperature in those quenches was very close to the transition point $T_{c}$ and possibly below it. The model suggests that quenching from a higher temperature would result in many more long polypeptides as possibly consistent with the fragmentary results from ocean trench measurements. The practical problem for a laboratory experiment is that to go to higher initial temperatures requires working at high pressures, so that the aqueous fluid does not boil. That brings more trouble and expense but is certainly doable in principle.

An alert reader will note that we have suggested two origins for a kind of critical temperature around the boiling point of water in this work. In this paper (11) and in [36], we have introduced a $T_{c}$ depending on kinetic factors including the distribution of activation energies for bond dissolution and the experimental time which the fluid spends at high temperature. In references [32,35] on the basis of the equilibrium distributions of polymer lengths at high and low temperatures, we suggested that a temperature near the boiling point of water at atmospheric pressure might arise because the equilibrium distribution of lengths would be nearly uniform at that temperature, leading to a wide distribution of lengths required by lifelike processes after quench. The latter temperature was estimated to be −Δ/lnb, where *b* is the number of types of available monomers and Δ is the equilibrium bond strength as defined above. It appears to be a coincidence that the two temperatures are close to one onother when b=20. However, it will not be hard to design experiments in which they are different, as indeed they are for example when b<20, and thereby to clarify the role of each.

I have emphasized protein synthesis leading to reproducing prion-like entities as a possible origin of life scenario because of its simplicity, although our models could be used with a mere change in parameters to study networks of nucleic acids. However, the parameters needed for such a simulation do not really favor a nucleic acid origin within our framework. In particular, the hydrolysis of nucleotide bonds is much more rapid in a prebiotic environment devoid of supporting proteins [50]. It is sometimes argued that the prion-like model cannot be right because there is no information-carrying molecule. However, I think that is to misunderstand the nature of information, which is essentially the negative of entropy up to an additive constant. The prion protein PrP ${}^{Sc}$, for example, is quite obviously carrying information in its secondary structure as evidenced by the fact that other varieties of the PrP protein with the same amino acid sequence but different secondary structures behave entirely differently and do not form reproducing prions. (Some work [51] has reported the use of data on secondary structures in modern biomolecules to construct a tree giving the history of terrestrial biological life. The results are interpreted as suggesting that proteins preceded nuclei acids in the history by several hundred million years).

If life originated in quenches of hot fluids from ocean rifts, then a much justified concern is that the fluids emitted would be rapidly dispersed from their origin by hydrodynamic processes, which would dilute them so much that any kind of lifelike process might be impossible. Examples of suggestions for trapping mechanisms which might prevent this are found, for example, in [52,53]. However, it appears that one might look for signs of prebiotic chemistry in contexts in which the bath of water into which the fluids were emitted was less turbulent and of smaller volume than around most ocean ridges. Existing ocean ridges approximating such conditions have been studied for example in the Bay of California [54] where the rift in the Guaymas Basin, unlike the Pacific ridges, is surrounded by detritus of biological origin on the ocean floor that is so deep that drilling projects have not yet been able to penetrate it to the bedrock beneath. The situation in the Red Sea [55] is reported to be similar. The age of the ocean floor around such slowly spreading rifts is believed to be too young to be the site of prebiotic chemistry. However, recent work [56] reports the discovery of more ancient material, up to nearly 3 billion years old, in and around such rifts. Whether its origin is from ancient continents or from recycling through the mantle after earlier subduction appears to be currently unknown. In any case, an ocean rift origin model suggests that the geological remanents of prebiotic evolution will be found in material near ancient slowly spreading rifts in ocean floors of several billion years ago, if such material can be identified.

## Data Availability

Data associated with the publications in references [20,30,31,32,35,36] has been or will shortly be publihed in the Data Repository of the University of Minnesota (DRUM) accessible at https://conservancy.umn.edu/handle/11299/166578.
